# Voicing demands for sex education and free contraception

**DOI:** 10.2471/BLT.16.040516

**Published:** 2016-05-02

## Abstract

Young people in Benin are starting to insist on their sexual and reproductive health rights, thanks to campaigners like Joannie Bewa. Dr Bewa talks to Fiona Fleck.

Q: How did you become interested in public health and why did you study medicine?

A: When I was 12, my best friend, Blandine, died from the complications of unsafe abortion. I was deeply shocked and her death inspired me to study medicine to try to improve the health of girls and women in my country. As part of my medical studies, I wrote a thesis on the cost of spontaneous, assisted and caesarian section deliveries. It’s shocking to see that women are still dying because their families can’t afford to pay for the delivery. In poor and rural areas, you often see families who can’t even afford to buy a bag of blood – that is enough to save the life of a woman who lost blood while giving birth.

Q: Why did you become a public health activist?

A: As a physician I am passionate about being able to diagnose and treat illness in my patients, but it’s even better if you can prevent these problems. Many people don’t realize that they can prevent health problems such as high blood pressure, diabetes, HIV (human immunodeficiency virus) infection, sexually transmitted diseases and malaria. Social inequalities, lack of education and poverty are often the realities determining people’s health and well-being. I realized that girls and women are still dying from preventable conditions in pregnancy or childbirth and that we need better data and better policies to stop these deaths. I studied hard every year to pass my medical exams the first time, so that I could devote my three months’ break to community volunteer work. At 17, I worked for the Beninese Association for Family Promotion, the International Planned Parenthood Federation chapter in Benin, as a volunteer, educating adolescents and young people about their sexual and reproductive health.

“My best friend, Blandine, died from the complications of unsafe abortion.”

Q: You and other campaigners created the Young Beninese Leaders’ Association when you were 21 years old. How did you do that?

A: When we created the youth group in 2010, we launched a campaign aimed at reaching 10 000 young people across the whole country. The slogan was “*sans capote, on est juste des potes*” in French, which means “without condoms, we are just friends”. We worked hard to made young people more aware that they need to use condoms to prevent unwanted pregnancies and to prevent HIV and other sexually-transmitted infections. We used awareness sessions, capacity building and various promotional activities including films, music events, soccer games, arts events and a writing contest. We knew we could not reach 10 000 people alone, so we partnered with the United States’ embassy, which helped to fund the campaign. In 2012, we decided to add leadership and entrepreneurship components to our youth group’s health programme and managed to get funding from the Young African Women Leaders’ Forum. We found that partnerships are the key to our group’s success.

Q: Which areas of health have you worked on most in your youth campaigns, who have you been targeting and with what messages?

A: Mainly sexual and reproductive health and rights, but also water and sanitation, infectious diseases such as malaria and Ebola and noncommunicable diseases such as high blood pressure and type 2 diabetes. We have two target groups: adolescents aged 15 to 19 years and people aged 20 to 35 years. We explain how to obtain condoms and other contraceptives, how to access sexual and reproductive health services and how to practise safe sex. We also train people to participate meaningfully in their communities to find solutions to the problems they face, like the lack of access to health services, lack of education opportunities and youth unemployment. We have organized several campaigns. In one of them, we demanded free health-care services for rural populations, especially children under five-years-old and pregnant women. In another campaign, we publicized the need to get regular blood pressure checks. In a campaign focused on HIV prevention, we handed out free condoms.

Q: What hurdles did you face as a woman campaigning for better health and health awareness in Benin?

A: I had to prove that our youth organization was able to mobilize young people and raise awareness on key issues. At the beginning, people didn’t understand why it was necessary to campaign in this way. Some people said our youth initiative wouldn’t achieve anything. I was one of the few young women activists campaigning to improve young peoples’ health. Activism and public service tend to be male dominated and, sometimes, I struggled to get funding and technical support for capacity building from local organizations. We needed more young women to raise their voices and talk about our issues. So I helped other young women make their volunteer work possible by giving them money from my scholarship grant or pocket money to pay for their transport or mobile phone bills. Not everyone was as lucky as I have been to have the support of their parents and mentors .

Q: As a health activist in Benin how have you collaborated with international organizations?

A: Last year I joined the new youth and adolescent section of the Partnership for Maternal, Newborn & Child Health (PMNCH) and attended the regional review of the *Global strategy for women's, children's and adolescents' health (2016–2030)*. I was part of the PMNCH delegation to the United Nations General Assembly in 2015, I’m a Women Deliver young leader, one of 200 young champions in maternal, newborn and child health from around the world. I recently joined the advocacy campaign to end child marriage and female genital mutilation with the African Union Commission and the United Nations Population Fund (UNFPA). At the Commission on Population and Development in New York in 2015, Benin was one of the few delegations to have a youth delegate as an official representative. With the support of UNFPA Benin, I joined the negotiations and contributed to the draft Benin statement, ensuring that it reflected young people’s concerns and that sexual and reproductive health issues were addressed in a progressive manner.

Q: How are you making the voices of young people heard in Benin?

A: Girls and women represent the majority of the population of Benin and are equally affected by economic, social, cultural, environmental and health-related challenges as men, but lack equal rights with men. The Young Beninese Leaders’ Association runs an awareness programme. The idea is to make young people – girls and boys and young men and women – more aware of their sexual and reproductive rights. We often hear stories from young women about how they became pregnant as adolescents because they were not educated about sexual matters and didn’t have access to contraceptives. If we want the next generation to achieve its full potential, we must invest more in girls’ education and women’s empowerment.

“We must invest more in girls’ education and women’s empowerment.”

Q: The people who have the power to decide on these matters are the politicians. How do you make the politicians more aware of the importance of these issues in Benin?

A: The Young Beninese Leaders’ Association joined a campaign led by an alliance of youth groups calling for sex education and free contraception. We got our messages across via traditional media – newspapers and radio – as well as social media. We wrote letters to politicians and other stakeholders and held meetings to discuss our demands with government health officials. Recently the health ministry proposed new legislation to provide comprehensive sex education and free contraception for the first time in our country. Hopefully this will become a reality in the near future.

Q: What is the Benin post-2015 campaign?

A: The Benin post-2015 campaign aims to increase participation by young people in Benin in the post-2015 development agenda of the United Nations. The UNFPA youth panel organized the official launch in Benin jointly with the Young Beninese Leaders’ Association. We held four e-consultations on Facebook and Twitter, carried out three field visits and attended national and global advocacy events such as the Commission on Population and Development. The post-2015 campaign in Benin has been a real success with 2000 young people contributing.

Q: How have you been involved in the post-2015 campaign?

A: I’ve been working on the implementation of the International Conference on Population and Development programme of action to make it reflect young people’s concerns better. The programme of action is a global framework developed by UNFPA to guide programmes and policies on people’s health and well-being. I have been helping to draft national youth policy for Benin, in which we advocate for free family planning services.

Q: What is your hope for the future in Benin and other African countries?

A: Thanks to our campaigns, young people in Benin are now talking more about the need for family planning and for youth friendly health services, in addition to traditional issues of concern such as employment and education. My hope is to see a new generation of African youth – young people who are educated, healthy and employed, and who live in an environment where no women die while giving birth, where pregnancies are planned and where health systems are strong.

